# Post-Operative Hemorrhage

**Published:** 1901-09

**Authors:** 


### Abstracts and Selections.

### Post=Operative Hemorrhage.

To the July 6th number of the *Journal of the American Medical Association* Dr. A. H. Cordier contributes an article on post-operative hemorrhage, in which he points out that the use of unstable ligature material on large pedicles and in locations where it is quickly absorbed, is responsible for many avoidable secondary hemorrhages. He has lost one patient from the slipping of a catgut ligature, and he knows of many similar cases. He now uses silk ligatures only. When hemorrhages occur in cases where the surgeon is in doubt, he thinks one or more stitches should be removed and the field of the operation exposed. It is never justifiable to readminister the anesthetic or to delay treatment longer than is absolutely necessary. After the hemorrhage is controlled, the judicious use of a deci-normal salt solution will do much to restore the patient.

The following conclusions are drawn: 1. In diagnosticating post-operative hemorrhage the operative history will aid much. 2. The symptoms of shock and those of hemorrhage are identical. 3. In suspected cases the cutting of a single stitch in the incision will tell. 4. The surgery must be quick and decisive in these cases. 5. In cases in which bleeding is likely to occur, a drainage tube should be used. 6. Large quantities of deci-normal salt solution will save many patients. This should be used both per rectum and by injection into the veins. 7. Strychnine, belladonna, etc., will not control bleeding from a uterine or ovarian artery any better than from any other artery. 8. The surgeon should do what his surgical conscience tells him is right. Late researches in hematology make it appear that an internal concealed hemorrhage may be demonstrated by a careful blood count. This, it is stated, will show a decrease in red cells and an increase in white cells. R.

